# Cholera-Morbus—Prize Offered by the Russian Government

**Published:** 1831-01-01

**Authors:** 


### XXIII.

### CHOLERA MORBUS.

THIS terrific epidemic has reached Astrachan, nay, even Moscow, and is menacing the Russian dominions in Europe. No doubt, it is despatched by Providence, as a substitute for WAR, which has so long ceased to thin the population of the world. The humane Autocrat, however, is not disposed to view the matter in this philosophic manner; and has liberally offered a reward of 25,000 rubles, £1100 sterling, for the best dissertation on the nature, cause, prevention, cure, &c. of cholera. It is not to be supposed that the immense mass of documents published—or rather collected by the Medical Boards of our three Indian Presidencies, together with the numerous monographs on the subject of cholera, should be known to the council of health in Russia. They are very imperfectly known even in this country. But be that as it may, while we admire the paternal solicitude and the munificent liberality of the Russian Emperor and government, in holding forth such a premium for the best dissertation on the disease in question, we venture to predict that not an IOTA of additional information to the stock on hand, will be thus elicited. In every parallel of latitude and longitude—and in every season of the epidemic, since its first commencement twelve years ago, the disease has been constantly modifying, if not changing its character, thus puzzling the etiologist, and embarrassing the practitioner! Under such circumstances, what is to be expected from a prize-dissertation? Twenty-five thousand rubles! What is to be gained by the Russian government from the best essay on cholera? The loss of the above sum—that's all.

